# Classical swine fever virus NS5A protein changed inflammatory cytokine secretion in porcine alveolar macrophages by inhibiting the NF-κB signaling pathway

**DOI:** 10.1186/s12985-016-0545-z

**Published:** 2016-06-14

**Authors:** Xiao-Ying Dong, Sheng-Qiu Tang

**Affiliations:** College of Yingdong Agricultural Science and Engineering, Shaoguan University, Daxue Road, Zhenjiang District, Shaoguan, 512005 China; North Guangdong Collaborative Innovation and Development Center for Swine Farming and Disease Control, Shaoguan, 512005 China

**Keywords:** CSFV, NS5A, Cytokine, RIG-I, NF-κB

## Abstract

**Background:**

Classical swine fever (CSF) caused by CSF virus (CSFV) is a highly contagious disease of the pigs. A number of studies have suggested that CSFV non-structural (NS) 5A protein is involved in CSFV-associated pathogenesis, but its mechanism is still uncertain. The aim of this study was to investigate the roles of NS5A protein in CSFV-associated pathogenesis in cultured porcine alveolar macrophages (PAMs).

**Methods:**

After PAMs cultured *in vitro* were transfected with CSFV NS5A, the alterations in IL-1β, IL-6 and TNF-α expression were determined by ELISA, the RIG-I signaling activity related to inflammatory cytokine secretion was investigated by Western blot and Immunofluorescent staining.

**Results:**

It was suggested that, the stable expressed CSFV NS5A solely had no influence on the expressions of inflammatory cytokines IL-1β, IL-6 and TNF-α in PAMs Moreover, NS5A protein could suppressed IL-1β, IL-6 and TNF-α expression induced by poly(I:C). It was also showed that NS5A protein did not impair the expressions of RIG-I, MDA5, IPS-1, NF-κB and IkBα in cells without poly(I:C) stimulation. Protein expressions of RIG-I, MDA5, IPS-1, NF-κB were not disrupted by NS5A protein in poly(I:C)-stimulated cells, while poly(I:C)-induced NF-κB nuclear translocation and activity was obviously suppressed by this protein. A suppression in poly(I:C)-induced IkBα degradation in NS5A-expressing cells was also observed.

**Conclusion:**

These data indicated that CSFV NS5A protein could inhibit the secretion of inflammatory cytokine induced by poly(I:C) through the suppression of the NF-κB signaling pathway, indicating the participation of CSFV NS5A protein in the pathogenesis of CSFV.

## Background

Classical swine fever virus (CSFV), the member of Flaviviridae family, causes heavily economic losses in pig industries [1]. The CSFV genome consists of a single large open reading frame (ORF) encoding a polyprotein of about 4,000 amino acids that is co- and posttranslationally processed by cellular and viral proteases, leading to at least 12 mature proteins- the structural proteins-core (C), E^rns^, E1 and E2, and the non-structural proteins -p7, NS2, NS3, NS4A, NS4B, NS5A and NS5B [2, 3]. Among these proteins, NS5A protein is receiving an increasing attention as a potential target for anti-CSFV therapy.

CSFV NS5A protein comprises 497 amino acids, and plays an important role in CSFV growth, viral RNA synthesis [4], induction of oxidative stress and inflammatory responses [5]. Furthermore, previous reports provided an insight into the mechanism by which CSFV NS5A could alter intracellular events associated with the viral infection. It was demonstrated that CSFV NS5A decreased internal ribosome entry site (IRES)-mediated CSFV translation in a dose-dependent manner, indicating that CSFV NS5A might play an important role in the switch from translation to replication in CSFV [6]. CSFV NS5A could contribute at least partially to modulation of CSFV replication through binding to a 5′untranslated region (UTR) or FKBP8 [7–9]. Our previous study also suggested that CSFV NS5A protein was involved in CSFV replication [10]. Hepatitis C virus (HCV) also belongs to the family of CSFV, and its protein NS5A has been intensely investigated. The mature HCV NS5A protein, generated by the action of the NS3/NS4A serine protease, is a phosphoprotein that exists in a basal or in a hyperphosphorylated state (p56 and p58) [11]. It has shown that HCV NS5A is an essential replicase component that can be complemented in trans [12, 13]. Mutations in HCV NS5A affected the rate of HCV replication, suggesting a role of HCV NS5A in modulating viral expression and replication [14]. Moreover, HCV NS5A was able to interfere with cellular proteins such as PI3K, p53, or Raf-1, enabling cell signal transduction in host to be regulated [15]. In the transgenic mouse model, it was discovered that HCV NS5A could impair both the innate and the adaptive immune response to promote chronic HCV infection [16]. The reports even suggested that HCV NS5A regulated cell cycle progression by modulating the expression of cell cycle regulatory genes [17].

Retinoic acid-inducible gene I (RIG-I) and melanoma differentiation-associated gene 5 (MDA5) are cytoplasmic DEx(D/H) box helicases that can detect intracellular viral products and transmit the signaling through interferon promoter-stimulating factor 1 (IPS-1) adaptor protein [18], which serves to activate multiple evolutionarily conserved signaling pathways, such as Interferons (IFNs), Nuclear Factor kB (NF-κB) and IFN-regulatory factors 3 (IRF3) [19]. Activation of these pathways often culminates in the induction of an array of antiviral and inflammatory cytokines, which are widely considered as crucial components of innate antiviral immunity [20, 21]. Although the signaling pathways such as MEK/ERK, PKR-p38 and p38MAPK regulated by HCV NS5A have been extensively characterized, so far little is known as to how CSFV NS5A may be linked with the NF-κB signaling and inflammatory cytokine expression. Therefore, in this paper, we took an investigation in the regulation mechanism of CSFV NS5A in poly(I:C)-induced inflammatory secretion in PAMs. The results provided for the first time evidence supporting the inhibitory role of CSFV NS5A in poly(I:C)-induced inflammatory secretion through the suppression of NF-κB translocation and activity, and IkBα degradation, which highlighted a potential mechanism of CSFV pathogenesis.

## Results

### CSFV NS5A protein down-regulated the secretion of inflammatory cytokines induced by poly(I:C) in PAMs

**Fig. 1:**
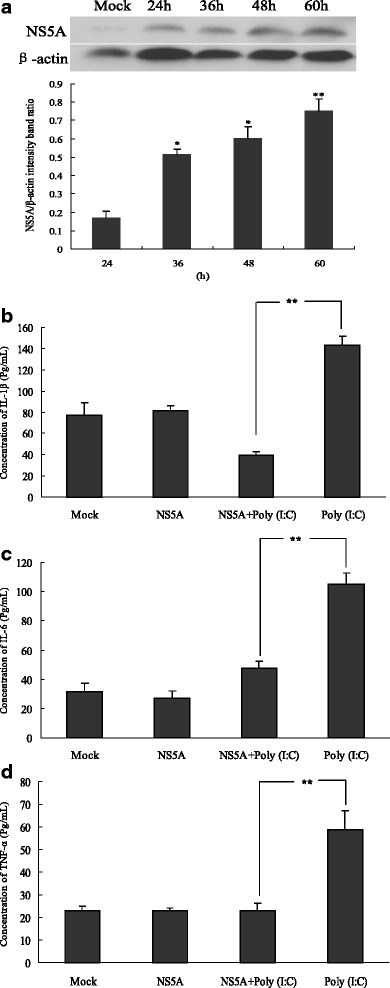
CSFV NS5A protein attenuated the inflammatory cytokine production induced by poly(I:C). PAMs were transfected with plasmid expressing NS5A (**a**). At indicated times, total cell lysates were immunoblotted with rabbit anti-CSFV sera and analyzed by Western Blot. To determine the changes of the inflammatory cytokine production in PAMs, at 24 h following transfection of 1 μg CSFV NS5A, cells were untreated or treated with 100 μg/mL poly(I:C), and cultured for 24 h. Then cell culture supernatants were collected to analyze protein expression of inflammatory cytokines IL-1β (**b**), IL-6 (**c**) and TNF-α (**d**) by ELISA. Data are expressed as mean ± SEM. Representative results are shown of one of three separate experiments. An asterisk indicates a statistically significant difference from uninfected cells, *P < 0.05 and **P < 0.01

The expression of the CSFV NS5A protein was analyzed by Western blot in PAMs. The results showed that, compared to the control without expressing NS5A gene (N1), CSFV NS5A protein was detectable after 24 h plasmid transfection in PAMs, and the size of protein was consistent with the expected size. Moreover, the expression of CSFV NS5A protein reached a maximum at 60 h (Fig. 1a).

At 24 h post-transfection, 100 μg/mL poly(I:C) was added into the cells for another 24 h and the impact of CSFV NS5A on endogenous inflammatory cytokine expression was examined using ELISA. It was suggested that 100 μg/mL poly(I:C) could significantly stimulate the secretion of IL-1β (Fig. 1b), IL-6 (Fig. 1c) and TNF-α (Fig. 1d) in PAMs. However, the over-expression of CSFV NS5A could significantly impair the secretion of IL-1β, IL-6 and TNF-α induced by poly(I:C) in the culture supernatant (*P* < 0.01). In addition, there was no difference in IL-1β, IL-6 and TNF-α expression between the control expressed vector and the CSFV NS5A-expressed treatment (*P* > 0.05), indicating that cytokine secretion was not affected in cells without poly(I:C) induction. Taken together, the results above suggested that CSFV NS5A protein had a strong inhibitory effect on the inflammatory responses induced by poly(I:C).

### CSFV NS5A protein showed no effects on the RIG-I/MDA5 signaling pathway in PAMs

**Fig. 2:**
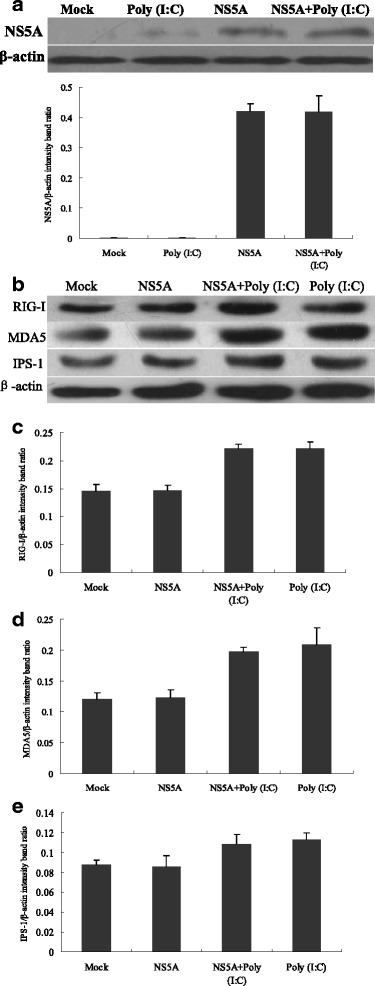
Changes of the RIG-I signaling pathway in CSFV NS5A-transfected cells. In PAMs, 1 μg CSFV NS5A was transfected for 24 h, then cells were untreated or treated with 100 μg/ml poly(I:C). After 24 h culture, extracts of circa 20 μg total cells were prepared and subjected to Western Blotting with antibodies specific for NS5A (**a**), RIG-I, MDA5 and IPS-1 in PAMs (**b**). Anti-β-actin was served as an internal control. Band ratio of RIG-I (**c**), MDA5 (**d**) and IPS-1 (**e**) was analyzed using Image J software. The experiment was repeated three times and the figure here shows a representative experiment. An asterisk indicates a statistically significant difference from uninfected cells, *P < 0.05 and **P < 0.01

To study the effects of CSFV NS5A protein on the RIG-I signaling pathway in greater detail, the protein expressions of RIG-I, MDA5 and IPS-1 were analyzed using Western Blot (Fig. 2b). It was indicated that, CSFV NS5A protein expression in PAMs was not changed by poly(I:C) stimulation (Fig. 2a). Compared to the control, a moderate higher expression of RIG-I, MDA5 and IPS-1 was appeared in NS5A transfected cells, but the effect was not significant (*P* > 0.05). Furthermore, the changes of RIG-I (Fig. 2c), MDA5 (Fig. 2d) and IPS-1 (Fig. 2e) expression were investigated after poly(I:C) stimulation. It was shown that poly(I:C) challenge significantly elevated RIG-I, MDA5 and IPS-1 production, and this effect was not affected by over-expression of CSFV NS5A (*P* > 0.05). Our results suggested that the CSFV NS5A had no influence on the RIG-I/MDA5 signaling pathway in PAMs with or without poly(I:C) stimulation.

### CSFV NS5A protein suppressed IkBα degradation induced by poly(I:C) PAMs

**Fig. 3:**
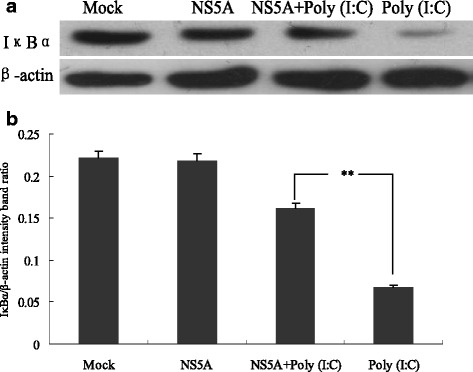
Changes of IkBα degradation in CSFV protein NS5A transfected cells. Cells were treated as demonstrated in Fig. 2. Expression of IkBα in PAMs was measured by Western Blotting with antibodies specific for IkBα (**a**), and analyzed using Image J software (**b**). The representative results are shown of one of three separate experiments. An asterisk indicates a statistically significant difference from uninfected cells, *P < 0.05 and **P < 0.01

Western Blotting was performed to detect the expression of IkBα which can keep NF-κB in inactivity in the cytoplasm (Fig. 3a). The results in Fig. 3b demonstrated that there was no significant change appeared in IkBα expression in CSFV NS5A-expressed PAMs in comparison with the control (*P* > 0.05), suggesting that CSFV NS5A protein did not alter the expression of IkBα. In contrast, cells treated with poly(I:C) showed a significant reduction of IkBα expression compared to that of a basal amount of IkBα in the control, suggesting that degradation of IkBα had occurred. Furthermore, IkBα degradation induced by poly(I:C) was rapidly suppressed in CSFV NS5A protein-treated cells (*P* < 0.01). The results above indicated that CSFV NS5A protein could inhibit poly(I:C)-induced IkBα degradation in PAMs.

### CSFV NS5A protein inhibited the activation of NF-κB/p65 in PAMs

**Fig. 4:**
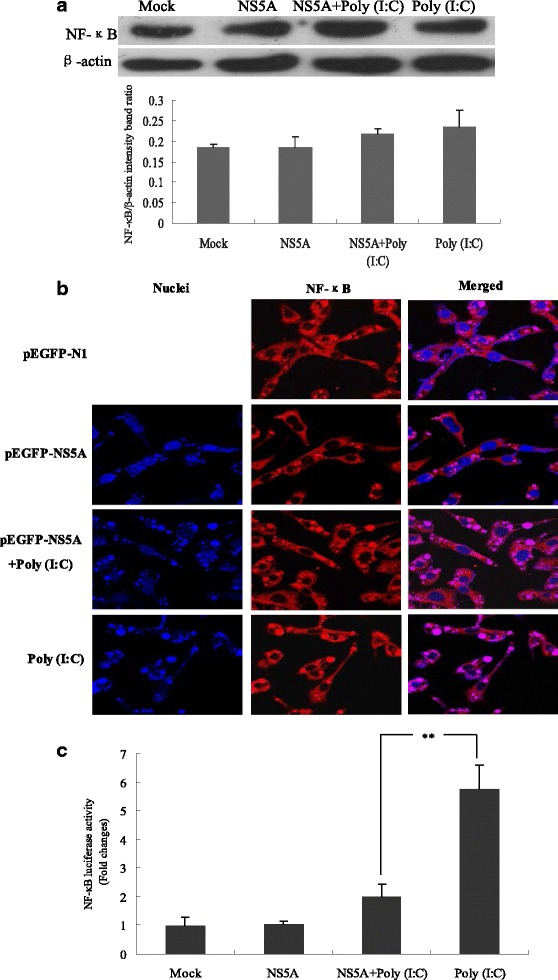
Effects of CSFV NS5A on the protein expression and nuclear translocation of NF-κB. Cells were treated as demonstrated in Fig. 2. Expression of NF-κB in PAMs was measured by Western Blot with antibodies specific for NF-κB (**a**). Cells were fixed and the localization of NF-κB (red) was observed by fluorescence microscope using immunofluorescence stain with anti-NF-κB/p65 and QDs-SA 605-conjugated biotinylated secondary antibodies. Nuclei were stained with DAPI. Bar, 10 μm (**b**). NF-κB Luciferase reporter assay was done to determine NF-κB luciferase activities (**c**). Representative results are shown of one of three separate experiments. An asterisk indicates a statistically significant difference from uninfected cells, *P < 0.05 and **P < 0.01

The production of pro-inflammatory cytokines and cellular adhesion molecules is controlled by the transcription factor NF-κB. To investigate whether the changes in IL-1β, IL-6 and TNF-α secretion induced by CSFV NS5A protein were associated with the activation of NF-κB signal, Western blot and Immunofluorescent staining assays were carried out to measure the expression and activity of the 65 kDa subunit of NF-κB, the results were shown in Fig. 4. As shown in Fig. 4a, a higher expression of NF-κB induced by poly(I:C) was not changed in CSFV NS5A-treated cells (*P* > 0.05). In addition, in control experiments, cells failed to signal NF-κB nuclear translocation, showing typical cytoplasmic staining of NF-κB. However, nuclear accumulation of NF-κB occurred within a larger frequency when cells were stimulated by poly(I:C) for 24 h, and these effects were obviously inhibited by CSFV NS5A, indicating that CSFV NS5A protein suppressed NF-κB nuclear translocation generated by poly(I:C) (Fig. 4b). Additionally, there was no difference in NF-κB luciferase activity between the control and CSFV NS5A-treated group (*P* > 0.05). But poly(I:C)-induced NF-κB Luciferase activity was significantly down-regulated by CSFV NS5A protein (*P* < 0.01) (Fig. 4c).

## Discussion

Classical swine fever (CSF) caused by CSF virus (CSFV) leads to severe economic losses in pig industry especially in developing countries. The role of CSFV NS5A on the molecular level has been well characterized, but much less is known about the relevance of CSFV NS5A for CSFV-associated pathogenesis. To gain more insight in CSFV NS5A protein, this study was conducted to explore the effect of CSFV NS5A on inflammatory cytokines and its mechanisms. Eventually, the results showed that, CSFV NS5A could suppressed poly(I:C)-stimulated inflammatory cytokine secretion by suppressing the NF-κB signaling pathway.

Following recognition of viral RNA, RIG-I and MDA5 undergo conformational changes for signal propagation to activate downstream through interactions with IPS-1 adaptor protein, which serves to activate downstream IRF, NF-κB and other transcription factors [22]. *In vitro* studies suggest that both RIG-I and MDA5 detect poly(I:C), a synthetic dsRNA analogue [23]. NF-κB, a sequence specific transcription factor, can regulate the expression of numerous cellular and viral genes and plays important roles in cell survival, tumorigenesis, inflammation and innate immune responses. In resting cells, NF-κB stays inactive in the cytoplasm combined with its inhibitory subunit IkBα. After exposure to a variety of agonists, the activation of NF-κB occurs through the degradation of IkBα [24, 25]. CSFV NS5A protein has shown to be involved in viral replication [7–9]. A closely related functional viral protein to the CSFV NS5A is the HCV NS5A protein while HCV belongs to the same Flaviviridae family. HCV NS5A is a remarkable protein as it clearly plays multiple roles in mediating viral replication, host-cell interactions and viral pathogenesis. Now, it is regarded as a new target for antiviral drugs in the treatment of HCV infection [26]. Recent reports have demonstrated that HCV NS5A protein exerts its functions through its regulation via cell signaling pathways such as STAT1 pathway [27], MEK/ERK pathway [28], a FoxO1-dependent pathway [29], and PKR-p38 pathway [30]. Furthermore, HCV NS5A over-expression significantly enhanced survivin transcription by increasing p53 degradation and stimulating NOS2A expression as well as NF-κB relocation to the nucleus [31]. HCV NS5A suppressed p53-mediated transcriptional transactivation and apoptosis during HCV infection [32], blocked poly(I:C) or interferon (IFN)-α-mediated IRF-7 nuclear translocation [33] or inhibited TNF-α-induced NF-κB activation *in vitro* [34]. Furthermore, HCV NS5A activated NF-κB through oxidative stress or tyrosine phosphorylation of IkBα and its degradation by calpain protease [35]. In the present study, we found that CSFV NS5A did not disrupt the expressions of RIG-I, MDA5, IPS-1 stimulated by poly(I:C) in PAMs. However, CSFV NS5A protein inhibited poly(I:C)-induced NF-κB nuclear translocation and activity, and IkBα degradation, which resulted in the suppression of inflammatory cytokine IL-1β, IL-6 and TNF-α secretion induced by poly(I:C).

Early detection of viruses by the innate immune system is critical for host defense. Antiviral immunity is first to be initiated by pattern recognition receptors (PRRs) that recognize viral pathogen-associated molecular patterns (PAMPs). Intracellular PRRs then stimulate the production of interferons and cytokines to orchestrate immune responses. The key host factors that are critical for antiviral immunity and for systemic inflammatory reactions include IL-1β, IL-6 and TNF-α [36]. TNF-a, IL-1 and IL-6 are three proinflammatory cytokines that form part of a complex defence network that protects the host against inflammatory agents, microbial invasion and injury [37]. IL secretion is necessary to stimulate immune cell responses and IL-1 is released from CSFV-infected macrophages [38]. Recent studies have demonstrated that the highly active proinflammatory cytokine IL-1β is essential in antiviral host defense. Despite its essential role in host defense, high levels of IL-1β are also responsible for unwanted effects like fever, vasodilatation, hypotension or acute lung injury by fluid accumulation in response to viral infection [39]. In the transgenic mouse model, HCV NS5A could impair both the innate and the adaptive immune response to promote chronic HCV infection [16] through the blockade of IFN-β induction by NS5B [40], the inhibition of interferon-alpha signaling [41], the competed binding to CypA [42], and a up-regulation of IL-8 [15]. The finding *in vivo* suggested that CSFV infection promoted serum levels of IFN-α, IL-8 and TNF-α in 6-month-old pigs, indicating the involvement of these cytokines in the immune response during CSFV infection with strains of different virulence [43]. Our previous study *in vitro* revealed that high virulent CSFV shimen strain could significantly promote the secretion of IFN-α, IFN-β, IL-1β, IL-6 and TNF-α through the activation of the RIG-I signaling pathway [44]. The present study further demonstrated that the stable expressed CSFV NS5A had no influence on the expressions of inflammatory cytokines IL-1β, IL-6 and TNF-α in PAMs without poly (I:C) stimulation. Moreover, CSFV NS5A protein could suppress IL-1β, IL-6 and TNF-α expression induced by poly (I:C).

## Conclusion

In summary, these findings provided novel information on the function of the poorly characterized CSFV NS5A and provided an insight into the mechanism by which CSFV NS5A could alter intracellular events associated with CSFV NS5A over-expression *in vitro*. It was suggested that CSFV NS5A could regulate poly(I:C)-stimulated inflammatory cytokine secretion by modulating the NF-κB signaling, which might help to find new approaches to prevent the establishment of a chronic CSFV infection.

## Methods

### Cell culture

Porcine alveolar macrophages (PAMs) were purchased from Cell Resource Center of Shanghai College of Health Sciences, Chinese Academy of Sciences (Shanghai, China). PAMs were maintained in RPMI 1640 supplemented with 10 % (vol/vol) fetal bovine serum (FBS), penicillin (100 units/mL), and streptomycin (100 mg/mL). All cells were cultured at 37 °C in a humidified 5 % CO_2_ incubator.

### Plasmid transfection

Plasmid pEGFP-NS5A was constructed in our laboratory. Approximately 1 × 10^6^ PAMs were plated into the well of a six-well tissue culture plate 24 h prior to transfection. Then cells were transfected with 1 μg pEGFP-N1 (the control without expressing NS5A gene) or pEGFP-NS5A. The Lipofectamine™2000 transfection reagent (Invitrogen, USA) was used for all transfection experiments. After 24, 36, 48 and 60 h transfection, the expression of NS5A protein was determined by Western Blot.

### Western Blot analysis

Western Blot analysis was carried out according to our previous study (Dong et al., 2013). In brief, six-well dishes of cells were transfected with pEGFP-N1 (the control) or pEGFP-NS5A plasmid at concentration of 1 μg for 24 h. Then cells were treated with 100 μg/mL poly(I:C). At indicated time periods, protein were extracted from cells, separated and transferred to the membranes. Following the incubation with primary antibodies monoclonal anti-MDA5 (1:1000, Sigma, USA), monoclonal anti-RIG-I (1:1000, Imgenex, USA), polyclonal anti-IPS-1 (1:400, Abgent, USA), polyclonal anti-NF-κB/p65 (1:1000, Thermo, USA), and polyclonal anti-IkBα (1:1000; Santa Cruz, USA), respectively, the membranes were washed and incubated with HRP-conjugated anti-rabbit secondary antibody (diluted 1/100000, Bioworld, USA). Then the membranes were developed with enhanced chemiluminescence (ECL) substrate (Beyotime, China) and exposed to X-ray film. As a control, gels were stripped and re-probed with antibody against monoclonal β-actin (1:1000, Beyotime, China) in this study. Band density was quantitated using Image J software.

### Immunofluorescent staining

In order to further verify the effects of NS5A on the nuclear accumulation of NF-κB, the subcellular localization of NF-κB in NS5A-expressing cells with or without poly(I:C) stimulation was examined by indirect immunofluorescence staining as demonstrated in our published article [44].

### ELISA

PAMs were seeded in six-well plates one day prior to virus infection and transfected with CSFV NS5A plasmid for 24 h. Then cells were treated with 100 μg/mL poly(I:C) (Sigma, USA) for 24 h. Cell culture supernatants were collected and used to analyze the production of IL-1β, IL-6 and TNF-α protein using enzyme-linked immunosorbent assays (ELISAs) kits (Uscn Life Science Inc, China) according to manufacturer’s protocols.

### NF-κB luciferase reporter assay

NF-κB Luciferase reporter assay was done as described in previous study [45]. To determine NF-κB luciferase activities, cells were infected with pNF-κB-luc (Beyotime, China) for 16 h. Then cells were transfected with NS5A plasmid for 24 h with/without poly(I:C). Cell protein were extracted using cell lysis buffer (Cell Signaling Technology, Danvers, MA, USA), and luciferase assays were performed using a Microplate Luminometer (Promega, Madison, WI, USA). Extract protein concentrations were normalized using Bio-Rad protein assay kits (Bio-Rad, Hercules, CA, USA).

### Statistical analysis

Results of the present study were analyzed by one-way analysis of variance and by Student’s t test with Bonferroni correction. All numerical data were collected from at least three separate experiments. Results were expressed as means ± standard deviation of the means. Results were considered statistically significant when a P value of less than 0.05 was obtained.

## Abbreviations

CSFV, Classical swine fever virus; ECL, Enhanced chemiluminescence; ELISAs, Enzyme-linked immunosorbent assays; FBS, Fetal bovine serum; HCV, Hepatitis C virus; IRF3, IFN-regulatory factors 3; IFN, Interferon; IPS-1, Interferon promoter-stimulating factor 1; IRES, Internal ribosome entry site; MDA5, Melanoma differentiation-associated gene 5; NS, Non-structural; NF-κB, Nuclear Factor Kb; ORF, open Reading frame; PAMPs, Pathogen-associated molecular patterns; PRRs, Pattern recognition receptors; PAMs, Porcine alveolar macrophages; RIG-I, Retinoic acid-inducible gene I; UTR, Untranslated region
