# Erratum to: Classical swine fever virus NS5A protein changed inflammatory cytokine secretion in porcine alveolar macrophages by inhibiting the NF-κB signaling pathway

**DOI:** 10.1186/s12985-016-0582-7

**Published:** 2016-08-03

**Authors:** Xiao-Ying Dong, Sheng-Qiu Tang

**Affiliations:** 1 College of Yingdong Agricultural Science and Engineering, Shaoguan University, Daxue Road, Zhenjiang District Shaoguan, 512005 China; 2 North Guangdong Collaborative Innovation and Development Center for Swine Farming and Disease Control, Shaoguan, 512005 China

## Erratum

Upon publication of this article [1], it was noticed that there had been an error in the processing of Fig. 4b. Although submitted correctly, the figure was published with the top left image missing. This has now been updated in the original article; please see the corrected figure below:

**Fig. 4:**
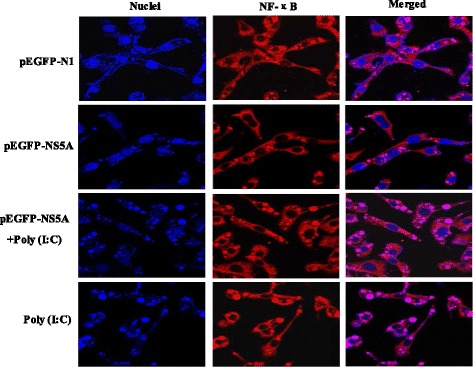
**b** NF-κB Luciferase reporter assay was done to determine NF-κB luciferase activities

## References

[1] Dong XY, Tang SQ. Classical swine fever virus NS5A protein changed inflammatory cytokine secretion in porcine alveolar macrophages by inhibiting the NF-κB signaling pathway. Virol J. 2016;13:101. 10.1186/s12985-016-0545-z PMC4907015 27296632

